# The mechanism of anti-tumor immunity induced by varlilumab, a CD27 agonist mAb, is model dependent

**DOI:** 10.1186/2051-1426-3-S2-P188

**Published:** 2015-11-04

**Authors:** Li-Zhen He, Anna Wasiuk, James Testa, Jeffrey Weidlick, Crsytal Sisson, Andrea Crocker, Jenifer Widger, Eirc Forsberg, Lauren Gergel, Lawrence Thomas, Henry Marsh, Tibor Keler

**Affiliations:** 1 Celldex Therapeutics, Inc., Hampton, NJ, USA; 2 Celldex Therapeutics, Inc., Needham, MA, USA

## 

The use of agonist antibodies to costimulatory receptors holds significant promise as a complementary approach to the already proven strategy of antibody mediated checkpoint blockade. This is in part due to the multiple mechanisms that have been associated with targeting costimulatory molecules in mouse models such as expansion of antigen-specific effector T cells (Teff), decrease in number or functional activity of regulatory T cells (Treg), and activation of NK cells. Recent reports have suggested that reduction of intratumoral Treg was particularly important for the anti-tumor activity of several immune modulating antibodies.

We have developed an agonist anti-human CD27 antibody, varlilumab, and have previously demonstrated its anti-tumor efficacy in several syngeneic tumor models using human CD27 transgenic mice. We set out to understand which mechanism of action of the CD27 antibody is related to its anti-tumor activity by developing mouse isotype variants of the antibody. Varlilumab expressed as a mouse IgG1 isotype (varli-mG1) induces potent costimulation and has no depleting activity, whereas, varlilumab expressed as a mouse IgG2a isotype (varli-mG2a) has moderate costimulatory activity and is capable of depleting cells with high CD27 expression (e.g. Treg). We found that as monotherapy, varli-mG1 was very effective in the BCL1 lymphoma model, and ineffective in the EG.7 thymoma model. In contrast, varli-mG2a was ineffective in the BCL1 model, but had anti-tumor activity in the EG.7 model. Based on correlative flow cytometry studies, we hypothesize that depleting Treg with high CD27 expression is the primary mechanism for anti-CD27 therapy in the EG.7 model, whereas the BCL1 model responds to potent CD27 costimulation. Interestingly, varlilumab (human IgG1 isotype) has a combination of the costimulation and Treg depleting activity and has potent anti-tumor activity in both models.

These data suggest that the mechanism of anti-tumor immunity induced by varlilumab is model dependent, and we are currently investigating this in additional tumor models. The results imply that multiple mechanisms are likely to also be relevant in the human setting. The preclinical studies and results from the Phase I trial with varlilumab are consistent with the antibody's capacity to mediate both T cell activation and a reduction in the number of Treg.

